# Using the theory of planned behaviour as a process evaluation tool in randomised trials of knowledge translation strategies: A case study from UK primary care

**DOI:** 10.1186/1748-5908-5-71

**Published:** 2010-09-29

**Authors:** Craig R Ramsay, Ruth E Thomas, Bernard L Croal, Jeremy M Grimshaw, Martin P Eccles

**Affiliations:** 1 Health Services Research Unit, University of Aberdeen, Foresterhill, Aberdeen, AB25 2ZD, UK; 2 Department of Clinical Biochemistry, Aberdeen University Medical School, Polwarth Building, Aberdeen, AB25 2ZD, UK; 3 Clinical Epidemiology, Ottawa Health Research Institute; Department of Medicine, University of Ottawa, Ottawa, Canada; 4 Institute of Health and Society, Newcastle University, Baddiley-Clark Building, Richardson Road, Newcastle upon Tyne, NE2 4AX, UK

## Abstract

**Background:**

Randomised trials of knowledge translation strategies for professional behaviour change can provide robust estimates of effectiveness, but offer little insight into the causal mechanisms by which any change is produced. To illustrate the applicability of causal methods within randomised trials, we undertook a theory-based process evaluation study within an implementation trial to explore whether the cognitions of primary care doctors' predicted their test requesting behaviours and, secondly, whether the trial results were mediated by the theoretical constructs.

**Methods:**

The process evaluation comprised a cross-sectional questionnaire survey of a random 50% sample of the randomised groups of primary care practices in Grampian (NHS Grampian), UK, who took part in a trial of the effect of enhanced feedback and brief educational reminders on test requesting behaviour. The process evaluation was based upon the Theory of Planned Behaviour and focussed on three of the test requesting behaviours that were targeted in the trial -- ferritin, follicle stimulating hormone (FSH), and Helicobacter Pylori serology (HPS).

**Results:**

The questionnaire was completed by 131 primary care doctors (56%) from 42 (98%) of the sampled practices. Behavioural intention, attitude, and subjective norm were highly correlated for all the tests. There was no evidence that perceived behavioural control was correlated with any of the other measures. Simple linear regression analysis of the rate of test requests on minimum behavioural intentions had R^2 ^of 11.1%, 12.5%, and 0.1% for ferritin, FSH, and HPS requesting, respectively. Mediational analysis showed that the trial results for ferritin and FSH were partially mediated (between 23% and 78% mediation) through intentions. The HPS trial result was not mediated through intention.

**Conclusions:**

This study demonstrated that a theory-based process evaluation can provide useful information on causal mechanisms that aid not only interpretation of the trial but also inform future evaluations and intervention development.

## Introduction

Randomised trials of knowledge translation (KT) strategies for professional behaviour change can provide robust estimates of effectiveness, but offer little insight into the causal mechanisms by which any change is produced. This would not be an issue if interventions had a uniform effect across different conditions that could be generalised to all practitioners outside of the trials. However, the effects of interventions do appear to vary by condition, professional group, and context, presumably because the causal mechanisms of the interventions are modified in the presence of different barriers and enablers [1]. Therefore the interpretation of a trial and assessment of its likely generalisability would be enhanced if additional information was obtained about the causal mechanisms through which the intervention worked, and how the effect was modified in the presence of different barriers and enablers.

There is increasing recognition of the value of process evaluations alongside trials of complex interventions such as professional behaviour change interventions. The behavioural sciences have developed and operationalised theories concerned with the determinants of behaviour and behaviour change [2]. These standard definitions of constructs and measurement methods may be useful for exploring causal mechanisms of interventions and barriers and enablers to KT. Theory-based process evaluations are a relatively new method being proposed to collect data on theoretical constructs alongside randomised trials to explore possible causal mechanisms [3]. This is akin to measuring intermediate endpoints in clinical trials to further understand the biological basis of any observed effects (for example, measuring cholesterol alongside trials of lipid-lowering drugs where the primary endpoint could be reduction in vascular related deaths). Different theories will be relevant to interventions at different levels; for example, psychological theories will likely be more relevant to interventions directed at individuals and teams, while theories of organisational change will be more relevant to interventions directed at hospitals or other large organisations.

We undertook a randomised controlled trial (RCT) [4] using a 2 × 2 factorial design to evaluate the effects of feedback of requesting rates enhanced with educational messages, and brief educational reminder messages, alone and in combination on UK primary care doctors' requesting of nine potentially overused laboratory tests. Practices that received either or both the enhanced feedback and the reminder messages were significantly less likely than the control group to request the targeted tests in total. The effect of the interventions varied across the targeted tests individually, although the general pattern showed a reduction in the number of tests requested for both interventions. Neither intervention was consistently better than the other. To investigate possible causal mechanisms, we conducted a post intervention survey using the theory of planned behaviour (TPB) about the use of three of the targeted laboratory tests -- the measurement of serum ferritin in the assessment of microcytic anaemia (ferritin), the measurement of serum follicle stimulating hormone (FSH) in the assessment of menopausal status, and the measurement of Helicobacter Pylori serology (HPS) following eradication therapy. Therefore, the aim of the study was to undertake a theory-based process evaluation study to explore whether the cognitions of general practitioners predicted their test requesting behaviours and secondly, whether the trial results were mediated by the theoretical constructs.

## Methods

### Description of the main trial interventions

Feedback consisted of a six-sided colour booklet (*e.g*., see Additional File 1) presenting graphs of practice level data for each of the nine targeted tests and for each laboratory discipline as a whole. Every graph showed rates of test requesting over the previous three years for the practice compared with the regional rates. The feedback was enhanced with brief educational messages that described specific clinical circumstances where it was inappropriate to request the test. These messages were included alongside the graphs for each of the targeted tests. The booklets were posted to each primary care doctor within each intervention group practice on four occasions (updated every three months from the start of the intervention period).

The brief educational messages were added as reminders to the test result reports sent to the requesting practice (*e.g*., see Additional File 2). The laboratory information system was programmed to recognise the relevant cues for each of the targeted tests and automatically add the brief educational reminder messages to the relevant printed and electronic test result reports. The messages were activated every time the cue occurred and were presented at the same time as the test result. The reminder messages were intended to influence future requests for the targeted tests.

### Choice of theory

**Figure 1:**
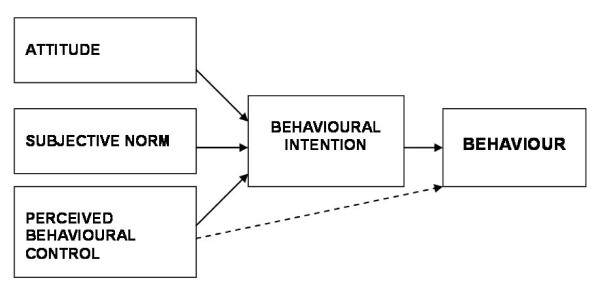
**The Theory of Planned Behaviour (Ajzen, 1991)**.

The process evaluation was based upon TPB (Figure 1) [5,6]. TPB is the social cognition model that has been widely used to predict individual behaviours [7,8] and has been one of the theories used most often when exploring the determinants of professional behaviour [9]. The theory states that an individual's intention to perform a behaviour is the proximal predictor of behaviour. In turn, intention is predicted by attitude (a person's overall evaluation of the behaviour), subjective norm (a person's own estimate of the social pressure to perform or not perform the target behaviour), and perceived behavioural control (the extent to which a person feels able to enact the behaviour; it has two aspects: how much a person has control over the behaviour and how confident a person feels about being able to perform or not perform the behaviour). Perceived behavioural control also has a direct effect on behaviour.

### Study design and population

The process evaluation comprised a cross-sectional questionnaire survey of a random 50% sample of the randomised groups of primary care doctors in Grampian (NHS Grampian), UK, who took part in a trial of the effect of enhanced feedback and brief educational reminders on test requesting behaviour. The random sampling was performed by a statistician independent of the research team.

We focussed the process evaluation on three of the test-requesting behaviours that were targeted in the trial -- serum ferritin, FSH, and HPS -- to reflect the varying effectiveness of the interventions. The choice of these tests reflected a range of effectiveness of the intervention. Whilst they were requested at similar rates prior to the intervention, following the intervention there were statistically significant reductions in the requesting of FSH, non-statistically significant reductions in Ferritin requesting and HPS requesting was unaffected.

### Data collection

#### Measures of the determinants of behaviour

We developed a direct measure TPB questionnaire to measure the determinants of the primary care doctors' test requesting behaviour [10]. We used standard worded items for each of four TPB constructs; intention, attitude, subjective norm, and perceived behavioural control, with three to five items contributing to each construct. The questionnaire was in three sections each corresponding to one of the three tests. At the start of each section, a brief scenario described the clinical problem that had been targeted by the brief educational messages. Primary care doctors were then asked to rate their intention, attitude, subjective norm, and perceived behavioural control related to requesting a specific test in the described scenario on a 7-point Likert scale (ranging from 1 'strongly agree' to 7 'strongly disagree'). The scenarios and examples of items (questions) for each construct are detailed in Table 1, and a full copy of the questionnaire can be seen in Additional File 3.

**Table 1:** Scenario details and examples of questionnaire items by construct

Scenarios	
**FSH: **Next week a woman aged 47 presents with hot flushes and night sweats having missed three of her last six periods (menopausal symptoms).	
**Helicobacter pylori serology: **Next week a patient comes to see you with symptoms of dyspepsia. You saw this patient three months ago, and prescribed antibiotics to eradicate helicobacter pylori which had been detected using a breath test	
**Ferritin: **Next week a patient returns to see you who presented complaining of tiredness. The result of his Full Blood Count (FBC) test shows a microcytic anaemia pattern (low MCV, low haemaglobin, low red cell count).	
	

**TPB Constructs**	**Example questions**
Behavioural intention (three questions)	I intend to request an FSH test to assess menopausal status in this woman - Strongly Agree/Strongly disagree
Attitudes (four questions)	I think that requesting a Helicobacter Pylori serology (HPS) test to assess efficacy of antibiotic therapy for eradication of helicobactor pylori in this patient is generally - Helpful/Unhelpful
Subjective norms (four questions)	Most general practitioners would request a Ferritin test to assess iron deficiency in this patient - Strongly Agree/Strongly disagree
Perceived behavioural control (five questions)	There are factors outside my control that would prevent me from requesting an FSH test for this patient - Strongly Agree/Strongly disagree.

The survey took place 12 months after the initiation of the interventions. A questionnaire plus reply paid envelope was posted to each primary care doctor, with one reminder sent to non-responders two weeks later.

#### Measures of behaviour

For each of the targeted test, the test requesting rate per 1,000 patients (standardised by practice list size) at 12 months post-intervention was used as the measure of behaviour in each primary care practice. The numbers of the three tests requested and the requesting practices for the 12 months of the intervention period were downloaded from the NHS Grampian laboratory information system. This data are routinely collected and ascribed to the primary care practice and could not accurately be attributed to individual primary care doctors.

### Statistical analysis

In all statistical analyses, the three targeted tests are reported and analysed separately. In order to test the representativeness of our sample from within the trial practices, the mean difference in test-requesting behaviour between sampled and non-sampled primary care practices was compared using a t-test.

### Scale generation

On the assumption that the tests were not necessary, responses for each of the four constructs were scaled from one to seven so that a high score on every construct (*e.g*. 7) equated with a low intention to request a test, a negative attitude towards requesting a test, a low perception of social pressure to request a test, and a high control over whether or not tests were requested.

For every primary care doctor, a score for each construct in the TPB model was calculated as the mean of all items contributing to the construct. Cronbach's alpha was used to ascertain the reliability of each of the scales. If reliability was lower than 0.7, an exploratory factor analysis was performed to identify any unreliable items and unreliable items were removed from the scale.

### Scale summaries

General descriptive statistics were used to summarise each scale and an intra-cluster correlation [11] was estimated to describe the degree of clustering of cognitions within each primary care practice. Pearson correlations were produced between all the scales. Multiple linear regressions of intention on attitude, subjective norm and perceived behavioural control were performed to identify significant predictors (2P < 0.05). To correct for the clustering within practice in the multiple regression models, the Huber-White estimator of variance inflation was used [12].

### Predicting test requesting using intention

To predict the strength of the relationship between intention and behaviour, because the behaviour data were at a practice level, a summary measure of intention for each practice had to be calculated. This was generated in two ways -- by taking the mean intention per practice (*i.e*., the average intention of all primary care doctors within a practice), and by taking the minimum intention per practice (*i.e*., the lowest intention score from any respondent within a practice). The minimum represented the lowest intention in each practice to do the correct behaviour (not request a test). The minimum was proposed as a possible summary measure because the severe negative skewness of the intention measures suggested that the poorest intention to perform the behaviour might be a better correlation with actual practice performance. Linear regressions were performed of behaviour on mean (or minimum) intention. For all analyses, effects were reported with corresponding 95% confidence intervals and the R^2 ^statistics were reported.

### Mediation analysis of trial result using intention

**Figure 2:**
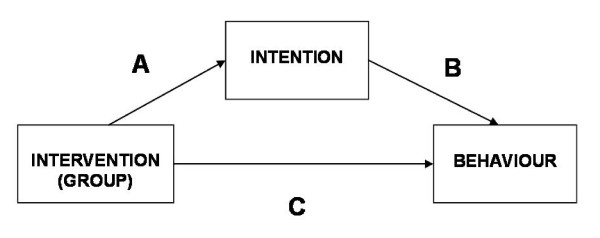
**Mediation Model - Intervention group as the predictor of behaviour, intention as the mediator**. The direct effect of the intervention allocation on behaviour is the coefficient **C **in the path diagram above. The indirect effect (often called the mediated effect) hypothesises that the observed intervention effect is due to a causal relationship whereby the intervention allocation "causes" the mediator variable (intention) to change and that in turn "causes" the behaviour to change. The indirect effect is therefore the product of the coefficients **A **and **B **in the statistical model and the direct effect is **C**. The strength of the mediation is determined by the difference between the direct minus indirect effect.

Summary descriptives of each TPB construct together with the behavioural outcome (test request rate per 1,000) were tabulated by randomised group. To estimate the strength of the mediation of intention on test requesting behaviour, a simple mediation model was setup with the trial group (reminders versus no reminders) as the predictor of behaviour and intention as the mediator (see Figure 2 Mediation Model). A bootstrapping method of estimating the indirect effect of intention was used [13], and the estimated percentage of the effect mediated through intention was reported. The same model was run for feedback versus no feedback.

## Results

### The survey sample

The questionnaire was sent to 232 primary care doctors in 43 practices. One hundred and thirty-one primary care doctors (56%) responded from 42 practices (33 primary care doctors from 10 control practices, 32 from 11 feedback practices, 31 from 10 reminders practices and 35 from 11 practices receiving both interventions). The mean requesting rate per 1,000 patients for each targeted test was similar in sampled and non-sampled practices (ferritin: 11.9 versus 15.8, p = 0.152; FSH: 10.1 versus 11.2, p = 0.474; HPS: 11.5 versus 11.5, p = 0.975).

### TPB constructs

The reliabilities of the behavioural intention, attitude, and subjective norm scales were greater than 0.70 (Table 2). For perceived behavioural control, exploratory factor analysis demonstrated that one question (how likely is it you would be able to request a ferritin/FSH/HPS in this patient?) was poorly correlated with the other items on the scale thereby reducing the reliability. When this item was removed, the reliabilities improved to the values shown in Table 2.

**Table 2:** Summary statistics of TPB construct scales across all respondents

N = 131	Mean	Median	SD	Minimum	Maximum	ICC	Reliability
**Ferritin**							
Behavioural intention	3.98	4.00	1.90	1.00	7.00	0.11	0.99
Attitude	3.97	4.00	1.63	1.00	7.00	0.16	0.97
Subjective norm	4.76	4.75	1.19	2.00	7.00	0.11	0.79
Perceived behavioural control	6.24	6.50	0.83	3.75	7.00	< 0.001	0.75
							
**FSH**							
Behavioural intention	5.07	5.83	1.66	1.00	7.00	0.17	0.99
Attitude	5.02	5.25	1.39	1.00	7.00	0.14	0.98
Subjective norm	4.86	5.00	1.17	2.00	7.00	0.03	0.82
Perceived behavioural control	6.14	6.37	0.86	2.75	7.00	0.08	0.70
							
**HPS**							
Behavioural intention	6.38	7.00	1.11	1.00	7.00	0.03	0.98
Attitude	6.26	7.00	1.17	1.00	7.00	0.15	0.92
Subjective norm	6.00	6.00	1.02	2.25	7.00	0.15	0.80
Perceived behavioural control	5.94	6.00	1.03	2.75	7.00	0.07	0.79

Summary statistics for each construct are shown in Table 2. Behavioural intention, attitudes, and subjective norms were at similar levels within ferritin test requesting (medians approximately equal to four) and similar within FSH test requesting (medians approximately equal to five). Perceived behavioural control had a median >6.0 for both ferritin and FSH test requesting. For HPS test requesting, all scales had median >6.0 suggesting a potential ceiling effect. Most of the intra-cluster correlations were >0.1, suggesting that primary care doctors had more similar cognitions to those in their own practice than to primary care doctors in other practices.

Behavioural intention, attitude, and subjective norm were highly correlated for all the tests (Table 3). There was no evidence that perceived behavioural control was correlated with any of the other measures. Multiple linear regression analyses demonstrated that attitude and subjective norm were predictive of intention for ferritin, FSH, and HPS requesting (Table 4). Perceived behavioural control was statistically significant for only the HPS requesting, but the R^2 ^for that model was lower than the others and intention had a clear ceiling effect suggesting the model fit was suboptimal and therefore unreliable.

**Table 3:** Correlations (Pearson's r) between TPB scales across all respondents

	Behavioural intention	Attitude	Subjective norm
**Ferritin**			
Attitude	0.91**	-	
Subjective norm	0.77**	0.76**	-
Perceived behavioural control	-0.11	-0.16	-0.01
			
**FSH**			
Attitude	0.91**	-	
Subjective norm	0.69**	0.60**	-
Perceived behavioural control	-0.07	-0.05	0.02
			
**HPS**			
Attitude	0.76**	-	
Subjective norm	0.73**	0.72**	-
Perceived behavioural control	-0.16	-0.10	-0.07

** Correlation was significant at 2P < 0.01

**Table 4:** Predictors of behavioural intentions and behaviour using multiple regression

	Coefficent* (95% CI)	p-value	**R**^**2**^
**Predictors of intentions**			
**Ferritin**			86%
Attitude	0.91(0.80, 1.02)	< 0.001	
Subjective norm	0.29 (0.11, 0.48)	< 0.01	
Perceived behavioural control	-0.08(-0.19, 0.04)	0.203	
			
**FSH**			86%
Attitude	0.94 (0.81, 1.06)	< 0.001	
Subjective norm	0.33 (0.14, 0.51)	< 0.01	
Perceived behavioural control	0.12 (-0.05, 0.28)	0.156	
			
**HPS**			65%
Attitude	0.46 (0.10, 0.81)	0.013	
Subjective norm	0.41 (0.12, 0.71)	0.008	
Perceived behavioural control	-0.10 (-.20, -.00)	0.048	
			

**Predictor of behaviour**			
**Ferritin**			
Intentions	-1.78 (-3.59, 0.03)	0.061	8.5%
**FSH**			
Intentions	-0.82 (-1.70, 0.06)	0.075	7.7%
**HPS**			
Intentions	0.28 (-1.98, 2.54)	0.808	0.1%

* Coefficient interpreted as the change in intentions (or behaviour) for each unit change in the construct (predictor)

### Predicting the rate of test requests

Simple linear regression analysis of the rate of test requests on mean behavioural intentions had R^2 ^of 8.5%, 7.7% and 0.1% for ferritin, FSH, and HPS, respectively Table 4). Replacing the mean intention per practice with the minimum intention per practice improved the R^2 ^to 11.1%, 12.5% and 0.1% for ferritin, FSH, and HPS, requesting respectively. The low R^2 ^for the HPS-requesting models was primarily due to a ceiling effect on intention generating little variability in the independent variables.

### Mediation analysis of trial result using intentions

Summary descriptives of each TPB construct are described by trial allocation in Table 5. For ferritin and FSH test requesting, there was a suggestion that the mean intention, attitude, and subjective norm differed between the intervention groups. For HPS requesting, all TPB constructs were skewed towards the positive end of the scales, suggesting very little variation between responses.

**Table 5:** Summary of scales and behaviour by trial allocation

	Behavioural Intention		Perceived Behavioural Control		Subjective Norm		Attitude		Rate per 1000 test requests	
	Median (IQR)		Median (IQR)		Median (IQR)		Median (IQR)		Mean (sd)	
Test Requested:										
**Ferritin**										
Control group	2.7	(2.0, 4.3)	6.3	(5.8, 7.0)	4.3	(3.5, 5.0)	3.25	(2.0, 4.0)	13.4	(6.7)
Feedback only group	4.3	(3.0, 6.0)	6.0	(5.3, 7.0)	5.0	(3.9, 5.8)	4.5	(3.6, 5.6)	11.8	(5.3)
Reminders only group	4.0	(2.1, 6.0)	7.0	(6.0, 7.0)	5.0	(4.0, 6.0)	4.0	(2.3, 5.2)	15.2	(14.6)
Both group	5.0	(2.9, 6.0)	6.3	(5.5, 7.0)	6.4	(5.8, 7.0)	4.5	(3.0, 5.3)	7.6	(3.6)
										
**FSH**										
Control group	4.3	(2.7, 6.0)	6.0	(5.8, 6.8)	4.2	(3.5, 5.2)	4.2	(3.2, 5.5)	11.3	(3.9)
Feedback only group	5.6	(4.3, 6.0)	6.5	(5.5, 7.0)	4.9	(4.2, 5.5)	5.2	(4.5, 6.0)	10.1	(2.7)
Reminders only group	6.0	(5.0, 7.0)	6.5	(6.0, 6.9)	5.2	(3.9, 5.9)	5.5	(4.7, 6.2)	9.6	(3.9)
Both group	6.0	(5.0, 7.0)	6.3	(5.5, 7.0)	5.2	(4.7, 5.9)	5.7	(4.9, 6.1)	9.5	(4.0)
										
**HPS**										
Control group	6.3	(6.0, 7.0)	5.8	(4.9, 6.5)	6.0	(5.5, 7.0)	7.0	(5.7, 7.0)	10.7	(4.9)
Feedback only group	7.0	(6.0, 7.0)	5.8	(5.0, 7.0)	6.0	(5.5, 6.0)	7.0	(6.0, 7.0)	13.6	(6.8)
Reminders only group	7.0	(6.0, 7.0)	6.8	(6.0, 7.0)	6.2	(5.0, 6.9)	7.0	(6.0, 7.0)	10.7	(4.6)
Both group	7.0	(7.0, 7.0)	6.0	(5.5, 7.0)	6.3	(5.8, 7.0)	7.0	(6.0, 7.0)	10.9	(5.9)

PBC = Perceived Behavioural Control

The results of the mediation analysis are shown in Table 6. The direct result was the trial effect (*i.e*., the difference in mean test requesting between the intervention and control groups). For example, reminders reduced the rate of ferritin test requesting by 1.33. Note in contrast to the main trial, none of the direct results were statistically significant because only 50% of practices were in the process evaluation. The indirect effect was the difference in trial effect of the intervention when behavioural intention was included and excluded from the model. For example, behavioural intention reduced the effect of reminders on ferritin tests by 0.39, resulting in 29% of the effect of the reminders being mediated through intention. For ferritin and FSH, there was some evidence that the trial effects were partially mediated by behavioural intentions. For HPS test requesting, there was a clear ceiling effect in behavioural intention making the mediation analysis unreliable.

**Table 6:** Mediational analysis of intentions on trial result

	Ferritin		FSH		HPS	
	Mean (95% CI)		Mean (95% CI)		Mean (95% CI)	
Main effect:						
**Reminders**						
Direct effect	-1.33	(-6.78, 4.11)	-1.11	(-3.35, 1.12)	-1.37	(-4.87, 2.13)
Indirect effect	-0.39	(-2.70, 1.22)	-0.86	(-2.53, 0.19)	0.21	(-.44, 1.47)
Percentage effect mediated by intentions	29%		77%		0%	
						
**Enhanced Feedback**						
Direct effect	-4.57	(-9.85, 0.70)	-0.66	(-2.91, 1.60)	1.55	(-1.94, 5.05)
Indirect effect	-1.31	(-3.66, 0.16)	-0.15	(-1.19, 0.50)	-0.10	(-1.44, 0.83)
Percentage effect mediated by intentions	28%		23%		0%	

## Discussion

This study demonstrated that TPB can be used as a tool for theory-based process evaluations with the aim of investigating possible causal mechanisms in KT intervention studies when the intervention is hypothesised to be mediated by the constructs of TPB. There were differences in intention, attitude, and subjective norm to FSH and ferritin test requesting, suggesting that the intervention may have enhanced attitudes and subjective norms resulting in higher intention and subsequent behaviour changes. Indeed, mediational analysis was highly suggestive that the differences in test requesting behaviour between trial groups were mediated through intention. There were high intentions, subjective norms, and attitudes for HPS requesting, suggesting that there may have been a psychological ceiling effect resulting in the observed lack of effect on test requesting behaviour in the trial.

This study had several strengths. First, the main trial demonstrated strong intervention effects (behaviour change), so provided an ideal platform to investigate why change did or did not occur. In particular, the randomisation element provided the opportunity to robustly investigate whether intentions mediated the trial result. Second, use of a well-established psychological model (TPB) enabled the psychological constructs to be clearly defined. Third, the derived measures of psychological constructs were sensitive to group allocation, suggesting that the constructs were identifying real differences. Finally, the TPB survey was returned completed from 42 of the 43 practices, suggesting that the results were generalisable.

The mediational analysis suggested that intentions to request an FSH or ferritin test were part of the causal pathway in the trial, *i.e*., the observed trial reduction in test requesting was partially mediated by a change in intentions. In our experience, formal mediational analyses have been rarely used to investigate the causal factors in KT randomised trials, and we suggest investigators should make more use of theory-based process evaluations.

Given that responses were received from several primary care doctors within a practice, we were able to demonstrate that there was clustering of psychological constructs within practices. Behavioural intentions and attitudes to test requesting had intra-cluster correlations greater than 0.1. This clustering provided some empirical evidence that social or organisational factors within practices may influence test-requesting behaviour. The clustering also needs to be considered from a statistical power perspective when conducting such surveys in the future. The effects of clustering are that precision is reduced and confidence intervals are wider than if clustering were not present. The surveys therefore need a larger sample size to attain the level of precision that investigators are interested in [14].

Whilst nearly all practices (42/43) were represented in the final survey, only 56% of the primary care doctors within those practices responded. This response rate from individual primary care doctors is very similar to that of other surveys of health professionals [15]. We cannot however be sure that the responders' views are representative of the practice, but the response rates were the same across the trial intervention groups suggesting that the results were not biased. Further, use of different measures of aggregated practice intention acted as a form of sensitivity analysis on the influence of different aggregation methods on the study results [16].

In this study, our behavioural outcome was practice level test requesting. Ideally, to operationalise TPB model faithfully, the outcome would be individual practitioner-level requesting. A multi-level model analysis could then be used to account for any clustering of behaviour or behavioural predictors by practice. However, it was not possible to obtain data on individual primary care doctors' requesting patterns from the administrative data systems. The implication for statistical analysis was that some measure of practice-level psychological cognitions had to be derived. An obvious summary measure is the mean cognition of the primary care doctors within each practice [16]. Using the mean cognition, intentions predicted about 8% of the variability in FSH and ferritin testing. Because the psychological measures were generally high with little variability, an alternative summary measure (the minimum) was considered. The minimum predicted about 12% of the variability in FSH and ferritin testing. The observed lack of relationship between HPS testing behaviour and intentions to request was likely due to the ceiling effect in intentions, but could also be due to the insensitivity in the behavioural measure. That is, whilst the intervention (and therefore the scenario description in the questionnaire) targeted requesting of the tests in specific clinical circumstances, the information system cannot distinguish between specific clinical circumstances (*e.g*., for HPS repeat testing after eradication therapy, the measure of behaviour was all HPS test requests because the information system cannot distinguish between initial tests, repeat-tests, and does not identify the reasons for the request). Therefore, our dependent variable may not exactly match the context of the intervention and scenario. Our findings and future investigations of causal mechanism would be strengthened by individual, context specific, measures of behaviour. We would recommend that researchers consider conducting a sensitivity analysis on any summary measure of psychological cognitions when attempting to describe a group level behaviour [16]

No formal statistical power calculation was performed for the survey. The confidence intervals for the testing of the constructs that were predictive of intentions (Table 3) and the models predicting behaviour-using intentions (Table 4) demonstrated the study was adequately powered to detect important effects. For the results of the mediational analysis the study was underpowered. This was due mainly to only 50% of the original study practices taking part in the survey. This meant that the original study findings on test-requesting behaviour could not be replicated with the same precision (though the magnitude of effects were similar). We would recommend that future studies of mediational factors in KT trials conduct a formal sample size to ensure adequate power for the theory based process evaluation.

We investigated behavioural predictors at one time point after initiation of the intervention *i.e*., the survey was conducted after the study interventions had been delivered for 12 months. In this example, the difference in constructs scores between intervention and control practices were large and provided evidence of changes in construct. Future process evaluations may be augmented by the addition of pre-intervention measures of behavioural predictors. Furthermore, the results of this study provide some evidence that TPB could be used to design an intervention. The ceiling effect on the intention to request HPS tests suggests that an intervention targeting a primary care doctors' intention to request the test would likely fail. In the context of the trial reported here, this would have suggested that feedback and reminders might not have been effective interventions to use and that was indeed the trial finding.

The aims of process evaluation alongside randomised trials of complex interventions are numerous (*e.g*., fidelity of implementation; mechanisms, mediators, and the process of change; acceptability) and often encompass a range of methods [17-19]. There are few RCTs of professional behaviour change strategies that utilise theory to investigate the process of change [20]. Whilst TPB seems to be the most commonly applied social cognition model for investigating health professional behaviour, few studies have attempted to predict clinical-related behaviour [9]. The results of this process evaluation utilising theory, re-enforces that TPB seem an appropriate theory to predict health professional behaviour change [9], and that it may offer useful insight into the processes underlying change (trial effects) in KT trials [17].

## Summary

Recognition of the KT gap has led to increased interest in more active KT strategies. Existing research demonstrates that professional behaviour change interventions can be effective, but the effectiveness of interventions appears to vary across different clinical problems, contexts, and organisations. This study demonstrated that a theory-based process evaluation can provide useful information on causal mechanisms that aid not only interpretation of the trial but also can inform future evaluations and intervention development. We encourage researchers to conduct and further develop methods for exploring causal mechanisms alongside rigorous evaluations of different strategies.

## Competing interests

MPE is an editor of Implementation Science, but has had no editorial responsibility for this manuscript. All other authors have stated no competing interests.

## Authors' contributions

All authors conceived the original trial. JMG and MPE conceived the theory based process evaluation. All authors contributed to the design of the study. CR and RT were responsible for running the project. CR was responsible for the statistical analyses. All authors interpreted the data and findings. CR wrote the first draft of the manuscript, all authors read and approved the final manuscript.

## Supplementary Material

Additional file 1 **Example of the feedback intervention**. Click here for file

Additional file 2 **Example of the reminders intervention**. Click here for file

Additional file 3 **The TPB questionnaire**. Click here for file
