# Supplementary material for: Using the theory of planned behaviour as a process evaluation tool in randomised trials of knowledge translation strategies: A case study from UK primary care
Source: Implement Sci. 2010 Sep 29;5:71. doi: 10.1186/1748-5908-5-71 (PMC2959079; doi:10.1186/1748-5908-5-71)
Supplement: Additional file 1 — Example of the feedback intervention. [file 1748-5908-5-71-S1.PDF]

Laboratory Requesting Rates & Educational Feedback

Month Year

Grampian University Hospitals

Laboratory Medicine

Practice Name
Address1
Address2
Address3

1 4 9 5

NHS
Grampian

This feedback of laboratory requesting patterns represents the first phase of a new initiative from the Laboratory Medicine Group aimed at both informing general practitioners about their use of laboratory services and providing educational information about particular tests that can cause confusion.

Educational feedback of this type will be sent to each practice every three months. We hope that this data and educational comments are helpful. Specific queries can be directed to:-

Dr Bernie Croal
Senior Lecturer/Consultant
Dept of Clinical Biochemistry
Aberdeen Royal Infirmary
Telephone: 01224-554098
email: b.croal@arh.grampian.scot.nhs.uk

The graphs below display the 6-monthly requesting pattern for your practice compared to the Grampian average (all rates adjusted to a practice list size of 10,000 patients) for three of the main laboratory areas. The pages following highlight similar requesting trends for specific tests where potential confusion in their indications for use or interpretation have been identified.

It must be stressed that these requesting trend comparisons are not an exact science, hence a requesting level above or below the Grampian average does not necessarily imply appropriate or inappropriate test use. Instead, specific sources of potential inappropriate test use have been identified and commented on. We hope that this type of educational feedback is found to be helpful.

Biochemistry Tests

6 monthly totals standardised to a practice list size of 10,000 patients.

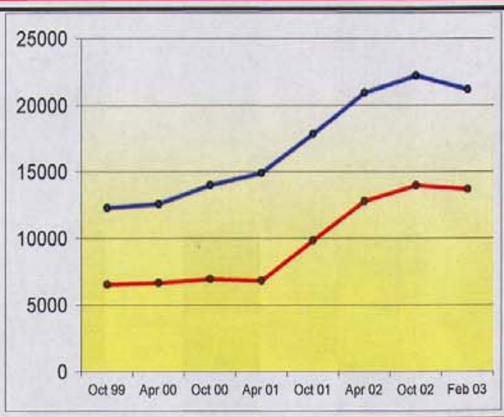

Grampian Average
21161.4

Your Practice
13684.0

Haematology Tests

6 monthly totals standardised to a practice list size of 10,000 patients.

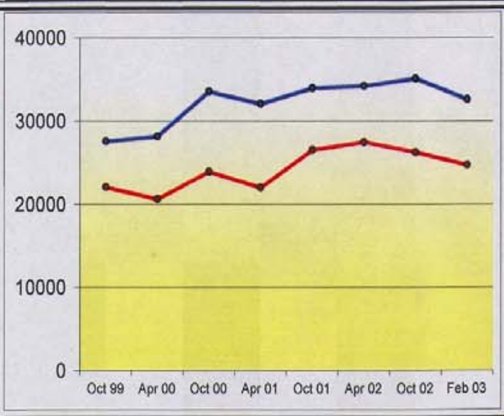

Grampian Average
32557.9

Your Practice
24718.8

Microbiology Tests

6 monthly totals standardised to a practice list size of 10,000 patients.

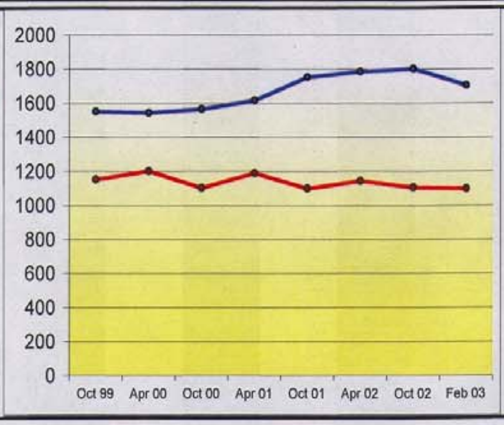

Grampian Average
1703.7

Your Practice
1098.9

Practice 0	N000

Carcino-Embryonic Antigen

Grampian Average	blue line	13.7
Your Practice	red line	3.7

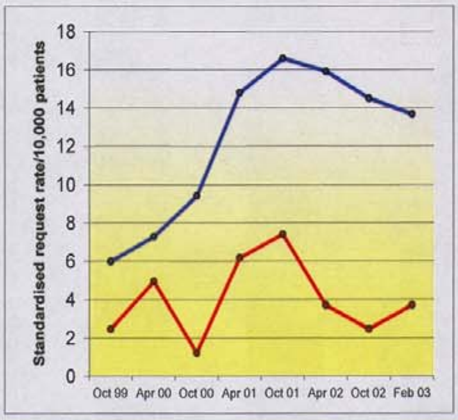

***Carcino-Embryonic Antigen (CEA) has been found to be raised in the serum of <u>some</u> patients with gastrointestinal malignancy, notably colonic cancer. It is therefore a useful marker for use in <u>monitoring</u> <u>treatment</u> and in detecting <u>recurrence.</u> CEA should <u>not</u> however be used to screen, diagnose or exclude malignancy. It can also be found to be raised in many other benign conditions, especially gastrointestinal disorders. False positive and false negative results can therefore lead to unnecessary further investigation or false reassurance.**

CA-125

Grampian Average	blue line	12.2
Your Practice	red line	1.2

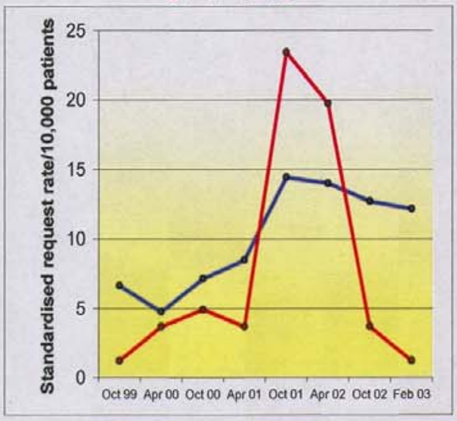

***CA-125 has been found to be raised in the serum of <u>some</u> patients with malignancy, notably ovarian cancer. It is therefore a useful marker for use in <u>monitoring</u> <u>treatment</u> and in detecting <u>recurrence.</u> CA-125 should not however be used to screen, diagnose or exclude malignancy. It can also be found to be raised in many other malignancies and benign conditions, notably gastrointestinal disorders. False positive and false negative results can therefore lead to unnecessary further investigation or false reassurance.**

Follicle Stimulating Hormone

Grampian Average	blue line	47.7
Your Practice	red line	40.7

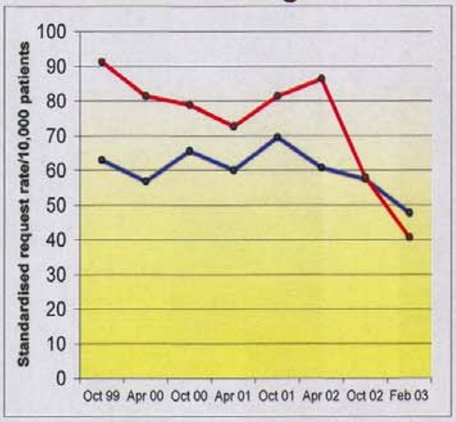

Follicle Stimulating Hormone (FSH) is released by the pituitary gland and acts to stimulate sex hormone production and reproductive processes. In general, FSH testing is of limited value in the assessment of menopausal status in women over 40 years of age, and so should <u>not</u> be requested for this purpose. Menopausal/Peri-menopausal status is best confirmed retrospectively based on clinical symptoms, signs and frequency or absence of menstruation. Biochemical measurement adds little to this classification, and may mislead.

Reading: Sturgeon C. Practice guidelines for tumour marker use in the clinic. Clinical Chemistry 2002; 48:

Practice 0	N000

Thyroid Stimulating Hormone

Grampian Average	—●—	689.9
Your Practice	—●—	431.7

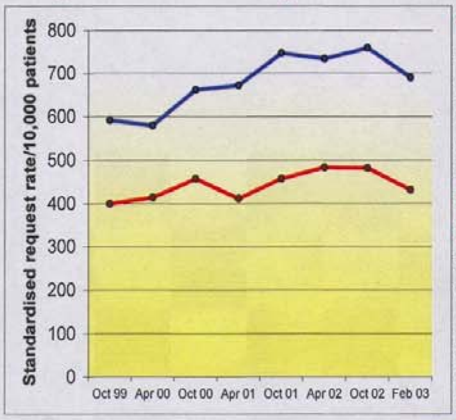

*Thyroid function tests (TFTs), including TSH are commonly used to screen for sub-clinical thyroid disease presenting as non-specific illness. In younger patients, as opposed to the elderly, thyroid disease rarely presents without specific clinical features. **TFTs are therefore not indicated as a screening procedure in young, clinically euthyroid patients.** In addition, TFT screening should be avoided in all patients with an acute illness of known cause unless intercurrent thyroidal disease is suspected. Any acute illness may cause thyroid hormone levels to become transiently abnormal.

Vitamin B12

Grampian Average	—●—	28.4
Your Practice	—●—	12.3

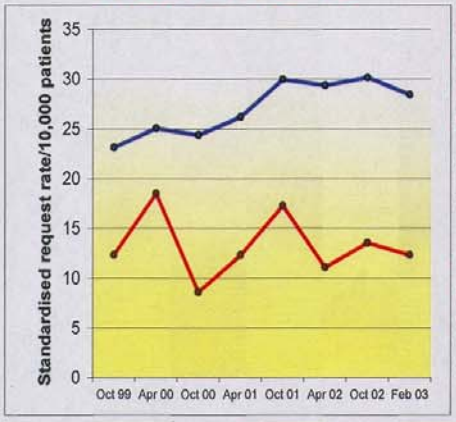

Macrocytosis without anaemia is unlikely to be due to B12 deficiency (thus should not be requested in the absence of anaemia). Thyroid or liver function tests may be helpful as they may signal an underlying cause for the macrocytosis. Alcohol abuse may also cause this picture.

Vitamin B12 levels are of no value and should therefore not be requested in patients undergoing parenteral B12 therapy; serum levels are generally always above the assay range in such patients receiving B12.

Ferritin

Grampian Average	—●—	66.3
Your Practice	—●—	54.3

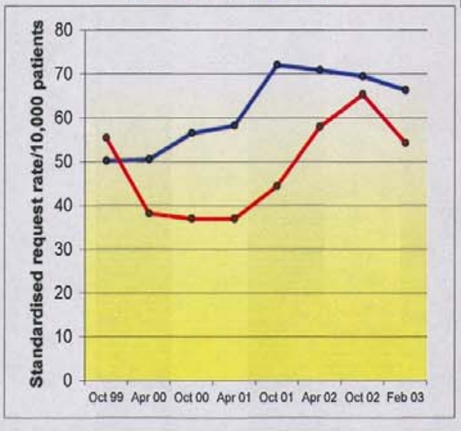

Ferritin measurement is generally unnecessary in caucasians with hypochromic microcytic anaemia as the underlying cause is almost always iron deficiency. While a source of potential blood loss should always be sought in cases of suspected iron deficiency, treatment with iron replacement is best monitored by clinical response and periodic haemoglobin measurement rather than resorting to iron studies - including ferritin assessment.

*Further Reading: O'Reilly D StJ. Thyroid function tests - time for a reassessment. BMJ 2000; 320: 1332-4

Autoantibody Screen

Grampian Average		31.2
Your Practice		14.8

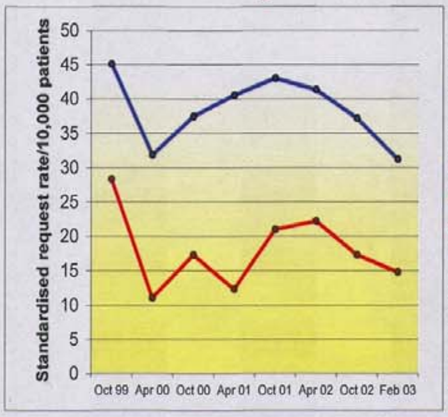

Detection of **autoantibodies** is a valuable aid to diagnosis of a range of autoimmune disorders. Chance detection of specific autoantibodies is however rarely of significant clinical value unless requesting is restricted to cases where there is a high clinical suspicion of autoimmunity. Autoantibody 'screen' requesting is inappropriate for investigation of non-specific illness. Requests should be test-specific; made only on the basis of specific clinical features. These tests are highly sensitive but have, in general, low diagnostic specificity, being found at significant frequency in non-autoimmune conditions such as infection, inflammation, malignancy, drug usage and in a proportion of the normal population (increasing with age).

IgE (Total/Specific)

Grampian Average		18.6
Your Practice		21.0

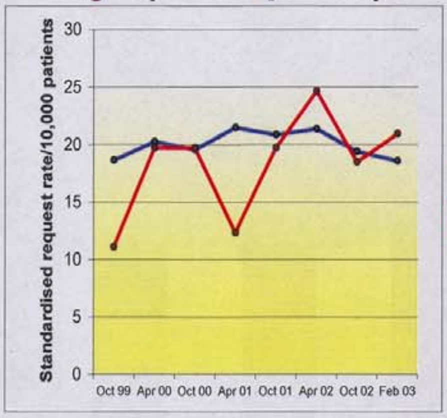

Raised total **IgE** and positive specific IgE tests are found in around 40% of the population but are only clinically relevant in around 20% (high false positive rate). General allergen "screening" is unhelpful. Allergen testing requests should instead be specific as directed by the history. IgE tests are not a substitute for adequate history taking. The significance of any result requires careful consideration in the context of the clinical problem.

Serum Helicobacter Pylori

Grampian Average		57.7
Your Practice		33.3

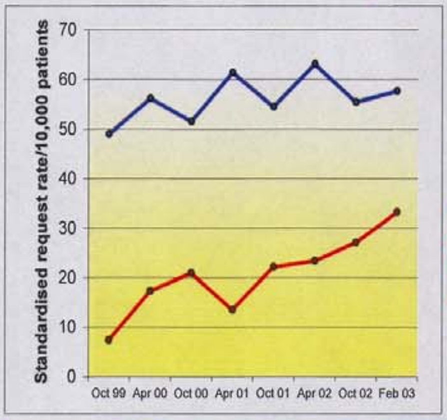

Helicobacter Pylori serology (**HPS**) and breath tests are highly sensitive markers of infection associated with symptomatic peptic ulcer disease. HPS should not be used to screen asymptomatic individuals, even if they have a family history of peptic ulcer disease. HPS should not be used to assess the efficacy of antibiotic eradication therapy as antibody levels may remain positive for some time after eradication. A Helicobacter pylori breath test should be instigated if peptic ulcer symptoms persist, so as to confirm/exclude continued infection.

*Further Reading: See Laboratory Handbook - Hard copy or via GUHT Intranet
