# Supplementary figures and images for: Using the theory of planned behaviour as a process evaluation tool in randomised trials of knowledge translation strategies: A case study from UK primary care
Source: Implement Sci. 2010 Sep 29;5:71. doi: 10.1186/1748-5908-5-71 (PMC2959079; doi:10.1186/1748-5908-5-71)

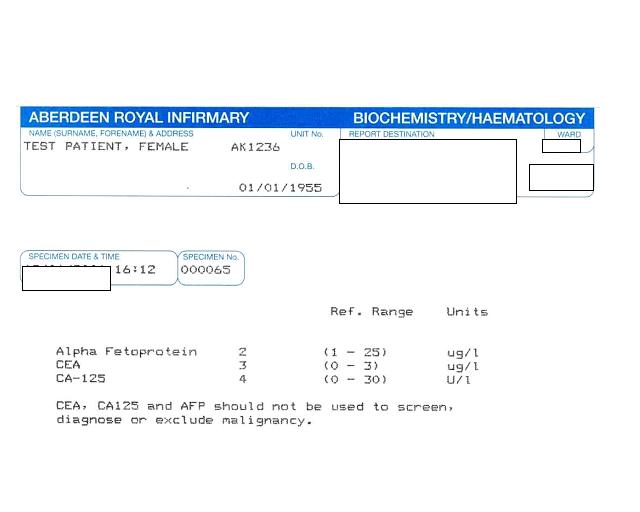

Supplement: Additional file 2 — Example of the reminders intervention. [file 1748-5908-5-71-S2.JPEG]
