# Supplementary material for: Using the theory of planned behaviour as a process evaluation tool in randomised trials of knowledge translation strategies: A case study from UK primary care
Source: Implement Sci. 2010 Sep 29;5:71. doi: 10.1186/1748-5908-5-71 (PMC2959079; doi:10.1186/1748-5908-5-71)
Supplement: Additional file 3 — The TPB questionnaire. [file 1748-5908-5-71-S3.PDF]

Code | | | |

DIAGNOSTIC REQUESTING ADVISORY MODEL EVALUATION

The purpose of this questionnaire is to find out your views and beliefs about requesting three particular laboratory tests within specific clinical situations. We appreciate that requesting these tests may be influenced by a range of factors. The questionnaire is designed to measure four factors:

- Doctors intentions to request the particular test
- Their attitude towards it
- How much social pressure they feel to request the test
- Whether they are actually able to request in their current clinical environment

There are between three and five questions for each measure. Some questions may therefore appear repetitive, this is necessary as previous research has found people respond differently to slightly different wording.

This questionnaire should take no more than 10 minutes to complete. **Please circle whichever number best describes what you think or your experience in the practice where you are working at the moment**. There are no right or wrong answers. Try not to take too long over each response – what comes to mind first is more likely to reflect what you believe.

Thank you very much for your time

Please return the questionnaire in the reply paid envelope provided. All information will be treated confidently.

If you wish to find out more about this study please contact either

Dr Bernie Croal
Senior Lecturer/Consultant
Dept of Clinical Biochemistry
Aberdeen Royal Infirmary
Tel: 01224-554098
Email: b.croal@arh.grampian.scot.nhs.uk

or

Dr Ruth Thomas
Health Services Research Unit
Polwarth Building
University of Aberdeen
Tel:01224 559296
Email r.e.thomas@abdn.ac.uk

**SCENARIO 1:**
Next week a woman aged 47 presents with hot flushes and night sweats having missed three of her last six periods (menopausal symptoms).

PLEASE CIRCLE RELEVANT RESPONSE

1. Attitude

I think that requesting a Follicle Stimulating Hormone (FSH) test to assess menopausal status in this woman is generally

Good practice	1	2	3	4	5	6	7	*Bad practice*
Helpful	1	2	3	4	5	6	7	*Unhelpful*
Appropriate	1	2	3	4	5	6	7	*Inappropriate*
Necessary	1	2	3	4	5	6	7	*Unnecessary*

2. Intention

I intend to request an FSH test to assess menopausal status in this woman.

Strongly agree	1	2	3	4	5	6	7	*Strongly disagree*

I would like to request an FSH test to assess menopausal status in this woman.

Strongly agree	1	2	3	4	5	6	7	*Strongly disagree*

I plan to request an FSH test to assess menopausal status in this woman.

Strongly agree	1	2	3	4	5	6	7	*Strongly disagree*

3. Social Pressure

Most general practitioners would request an FSH test to assess menopausal status in this woman.

Strongly agree	1	2	3	4	5	6	7	*Strongly disagree*

In general, I feel under pressure from patients to order a test to assess menopausal status.

Strongly agree	1	2	3	4	5	6	7	*Strongly disagree*

People who are important to me think that I should request an FSH to assess menopausal status.

Strongly agree	1	2	3	4	5	6	7	*Strongly disagree*

The RCGP would approve of requesting an FSH to assess menopausal status in this patient.

Strongly agree	1	2	3	4	5	6	7	*Strongly disagree*

4. Ability

Requesting an FSH test for this patient is:

Very easy	1	2	3	4	5	6	7	*Very difficult*

I am confident that I could request an FSH for this patient if I wanted to.

Strongly agree	1	2	3	4	5	6	7	*Strongly disagree*

There are factors outside my control that would prevent me from requesting an FSH test for this patient.

Strongly agree	1	2	3	4	5	6	7	*Strongly disagree*

I have complete control over whether to request an FSH test to assess menopausal status in this woman?

Strongly agree	1	2	3	4	5	6	7	*Strongly disagree*

How likely is it that you would be able to request an FSH test for this woman?

Very likely	1	2	3	4	5	6	7	*Very unlikely*

Please add any comments about any factors that make it difficult or easy to request an FSH test in this clinical situation

Difficult

Easy

SCENARIO 2:
Next week a patient comes to see you with symptoms of dyspepsia. You saw this patient three months ago, and prescribed antibiotics to eradicate helicobacter pylori which had been detected using a breath test.

PLEASE CIRCLE RELEVANT RESPONSE

1. Attitude
I think that requesting a Helicobacter Pylori serology (HPS) test to assess efficacy of antibiotic therapy for eradication of helicobactor pylori in this patient is generally

Good practice	1	2	3	4	5	6	7	*Bad practice*
Helpful	1	2	3	4	5	6	7	*Unhelpful*
Appropriate	1	2	3	4	5	6	7	*Inappropriate*
Necessary	1	2	3	4	5	6	7	*Unnecessary*

2. Intention
I intend to request an HPS test to assess efficacy of treatment in this patient.

Strongly agree 1 2 3 4 5 6 7 *Strongly disagree*

I would like to request an HPS test to assess efficacy of treatment in this patient.

Strongly agree 1 2 3 4 5 6 7 *Strongly disagree*

l plan to request an HPS test to assess efficacy of treatment in this patient.

Strongly agree 1 2 3 4 5 6 7 *Strongly disagree*

3. Social Pressure
Most general practitioners would request an HPS test to assess efficacy of eradication therapy for H pylori.

Strongly agree 1 2 3 4 5 6 7 *Strongly disagree*

In general, I feel under pressure from patients to order a test to assess efficacy of treatment of H-pylori.

Strongly agree 1 2 3 4 5 6 7 *Strongly disagree*

People who are important to me think that I should request an HPS test to assess efficacy of treatment of H-pylori.

Strongly agree 1 2 3 4 5 6 7 *Strongly disagree*

The RCGP would approve of requesting an HPS test to assess efficacy of treatment of H-pylori.

Strongly agree 1 2 3 4 5 6 7 *Strongly disagree*

4. Ability

Requesting an HPS test to assess efficacy of treatment in this patient is:

Very easy	1	2	3	4	5	6	7	*Very difficult*

I am confident that I could request an HPS test for this patient if I wanted to do

Strongly agree	1	2	3	4	5	6	7	*Strongly disagree*

There are factors outside my control that would prevent me from requesting an HPS test for this patient

Strongly agree	1	2	3	4	5	6	7	*Strongly disagree*

I have complete control over whether to request an HPS test to assess efficacy of treatment in this patient

Strongly agree	1	2	3	4	5	6	7	*Strongly disagree*

How likely is it that you would be able to request an HPS test for this patient?

Very likely	1	2	3	4	5	6	7	*Very unlikely*

Please add any comments about any factors that make it difficult or easy to request an HPS test in this clinical situation.

Difficult

Easy

SCENARIO 3:
Next week a patient returns to see you who presented complaining of tiredness. The result of his Full Blood Count (FBC) test shows a microcytic anaemia pattern (low MCV, low haemaglobin, low red cell count).

PLEASE CIRCLE RELEVANT RESPONSE

1. Attitude
I think that requesting a Ferritin test to assess iron deficiency in this patient is generally

Good practice	1	2	3	4	5	6	7	*Bad practice*
Helpful	1	2	3	4	5	6	7	*Unhelpful*
Appropriate	1	2	3	4	5	6	7	*Inappropriate*
Necessary	1	2	3	4	5	6	7	*Unnecessary*

2. Intention
I intend to request a Ferritin test to assess iron deficiency in this patient.

Strongly agree 1 2 3 4 5 6 7 *Strongly disagree*

I would like to request a Ferritin test to assess iron deficiency in this patient.

Strongly agree 1 2 3 4 5 6 7 *Strongly disagree*

I plan to request a Ferritin test to assess iron deficiency in this patient.

Strongly agree 1 2 3 4 5 6 7 *Strongly disagree*

3. Social Pressure
Most general practitioners would request a Ferritin test to assess iron deficiency in this patient.

Strongly agree 1 2 3 4 5 6 7 *Strongly disagree*

In general, I feel under pressure from patients to order a test to assess to iron deficiency.

Strongly agree 1 2 3 4 5 6 7 *Strongly disagree*

People who are important to me think that I should request a Ferritin test to assess iron deficiency in this patient.

Strongly agree 1 2 3 4 5 6 7 *Strongly disagree*

The RCGP would approve of requesting a Ferritin test to assess iron deficiency in this patient.

Strongly agree 1 2 3 4 5 6 7 *Strongly disagree*

4. Ability

Requesting a Ferritin test to assess iron deficiency in this patient is:

Very easy	1	2	3	4	5	6	7	*Very difficult*

I am confident that I could request a Ferritin test for this patient if I wanted to.

Strongly agree	1	2	3	4	5	6	7	*Strongly disagree*

There are factors outside my control that would prevent me from requesting a Ferritin test for this patient.

Strongly agree	1	2	3	4	5	6	7	*Strongly disagree*

I have complete control over whether to request a Ferritin test to assess iron deficiency in this patient

Strongly agree	1	2	3	4	5	6	7	*Strongly disagree*

How likely is it that you would be able to request a Ferritin test to assess iron deficiency in this patient?

Very likely	1	2	3	4	5	6	7	*Very unlikely*

Please add any comments about what factors make it difficult or easy to request a Ferritin test in this clinical situation

Difficult

Easy

Any other comments you would like to make

Thank you for your time
